# 5-HT receptors mediate lineage-dependent effects of serotonin on adult neurogenesis in *Procambarus clarkii*

**DOI:** 10.1186/1749-8104-6-2

**Published:** 2011-01-04

**Authors:** Yi Zhang, Jeanne L Benton, Barbara S Beltz

**Affiliations:** 1 Neuroscience Program, Wellesley College, Wellesley, MA 02481, USA

## Abstract

**Background:**

Serotonin (5-HT) is a potent regulator of adult neurogenesis in the crustacean brain, as in the vertebrate brain. However, there are relatively few data regarding the mechanisms of serotonin's action and which precursor cells are targeted. Therefore, we exploited the spatial separation of the neuronal precursor lineage that generates adult-born neurons in the crayfish (*Procambarus clarkii*) brain to determine which generation(s) is influenced by serotonin, and to identify and localize serotonin receptor subtypes underlying these effects.

**Results:**

RT-PCR shows that mRNAs of serotonin receptors homologous to mammalian subtypes 1A and 2B are expressed in *P. clarkii *brain (referred to here as 5-HT_1α _and 5-HT_2β_). *In situ *hybridization with antisense riboprobes reveals strong expression of these mRNAs in several brain regions, including cell clusters 9 and 10 where adult-born neurons reside. Antibodies generated against the crustacean forms of these receptors do not bind to the primary neuronal precursors (stem cells) in the neurogenic niche or their daughters as they migrate, but do label these second-generation precursors as they approach the proliferation zones of cell clusters 9 and 10. Like serotonin, administration of the *P. clarkii *5-HT_1α_-specific agonist quipazine maleate salt (QMS) increases the number of bromodeoxyuridine (BrdU)-labeled cells in cluster 10; the *P. clarkii *5-HT_2β_-specific antagonist methiothepin mesylate salt (MMS) suppresses neurogenesis in this region. However, serotonin, QMS and MMS do not alter the rate of BrdU incorporation into niche precursors or their migratory daughters.

**Conclusion:**

Our results demonstrate that the influences of serotonin on adult neurogenesis in the crayfish brain are confined to the late second-generation precursors and their descendants. Further, the distribution of 5-HT_1α _and 5-HT_2β _mRNAs and proteins indicate that these serotonergic effects are exerted directly on specific generations of neuronal precursors. Taken together, these results suggest that the influence of serotonin on adult neurogenesis in the crustacean brain is lineage dependent, and that 5-HT_1α _and 5-HT_2β _receptors underlie these effects.

## Background

The monoamine neurotransmitter 5-hydroxytryptamine (5-HT, serotonin) is found in the nervous systems of all organisms and is known to influence diverse physiological, behavioral and cognitive functions [1]. Among these actions, serotonin is a potent regulator of cell division, including the cell cycle of neuronal precursors in the adult brain [2-4].

**Figure 1:**
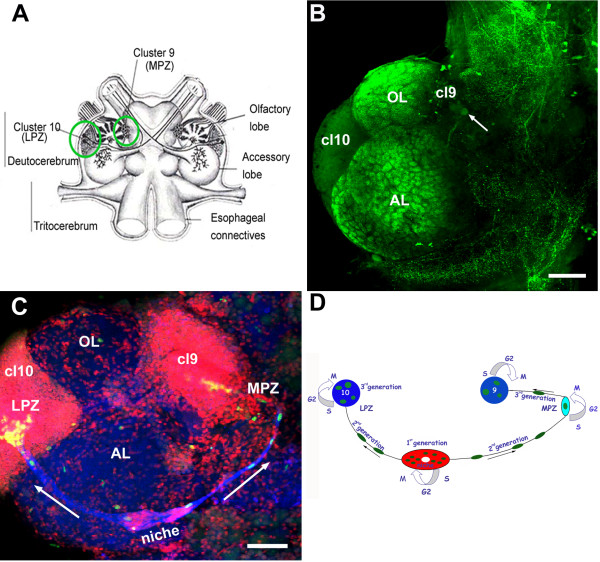
**The neurogenic system in the adult crayfish brain**. **(A) **Diagram of the crayfish brain. The soma clusters 9 and 10 (circled) flank two prominent neuropil regions of the deutocerebrum, the olfactory (OL) and accessory (AL) lobes. The OL is the primary olfactory processing area in the crustacean brain and is functionally equivalent to the olfactory bulb. The AL is a higher order processing area that integrates olfactory, visual and mechanosensory information [8,9]. **(B) **Serotonin immunostaining of a crayfish brain. Each dorsal giant neuron (DGN; arrow) innervates the ipsilateral OL and AL. The intense labeling of the OLs and ALs is due to the massive DGN projection to these areas. (cluster 9, cl9; cluster 10, cl10) **(C) **Confocal image of the ventral surface of the crayfish *P. clarkii *brain labeled immunocytochemically for 5-bromo-2-deoxyuridine (BrdU; green) and glutamine synthetase (blue), and counterstained with propidium iodide (red), a marker of nucleic acids. BrdU-labeled cells can be observed in the migratory streams (long arrows indicate direction of movement) and within both the lateral proliferation zone (LPZ) and medial proliferation zone (MPZ). The LPZ supplies cells to cluster 10, where they will differentiate into olfactory projection neurons. The MPZ supplies cells to cluster 9, where they will differentiate into local olfactory interneurons. **(D) **Model summarizing our current view of events leading to the production of new olfactory interneurons in adult crayfish. Neuronal precursor (first generation) cells exhibiting glial characteristics reside within a neurogenic niche where they divide symmetrically. Their daughters (second-generation precursors) migrate along tracts created by the fibers of the niche cells towards either the LPZ or the MPZ. At least one more division will occur in the LPZ and MPZ before the progeny (third- and subsequent-generations) differentiate into neurons. Scale bars: 200 μm (B); 100 μm (C). Panels (A, D) adapted from Sullivan *et al*. [11].

Adult neurogenesis, the production of functionally integrated neurons in the juvenile and adult brain, is a common feature in a variety of species, from insects and crustaceans to birds and mammals [5]. Throughout their lives, many decapod crustaceans add new interneurons to olfactory processing areas in the brain [2,6] that receive dense serotonergic innervation [7-9] (Figure 1A, B). In crayfish, adult neurogenesis involves at least three generations of precursor cells [10,11]. The primary (first generation) precursor cells reside in a vascularized niche (Figure 1C, D). These bipolar niche cells also provide a tract along which their progeny migrate. These second-generation migratory precursors move towards the medial proliferation zone (MPZ) and lateral proliferation zone (LPZ) of cell clusters 9 and 10 (terminology of Sandeman *et al*. [12]), where they divide at least once more. Their progeny differentiate into cluster 9 (local) and 10 (projection) olfactory interneurons, respectively [13].

Several lines of evidence indicate that serotonergic pathways influence adult neurogenesis in decapod crustaceans. Firstly, chronic depletion of serotonin with the pharmacological agent 5,7-dihydroxytryptamine attenuates neurogenesis [3,14]. Secondly, the rate of neurogenesis is highly dependent on serotonin concentration [15]. Finally, electrical activation of one of the paired serotonergic dorsal giant neurons (DGNs) causes a tenfold elevation in serotonin levels and significant increases in neurogenesis in the ipsilateral cluster 10, compared with levels of neurogenesis in cluster 10 on the unstimulated, contralateral side of the same brain [16].

Serotonin mediates physiological functions in vertebrates and invertebrates by activating diverse receptors. In mammals, seven classes of serotonin receptors comprising at least 15 subtypes, defined by their signal transduction mechanisms and pharmacological properties, have been identified [17,18]. In arthropods, it is predicted that at least 18 monoamine receptors exist [19], and in crustacean species serotonin receptors account for 5 or more of these [20-22]. However, only two crustacean serotonin receptors have been cloned and characterized, and these are homologous to vertebrate subtypes 1A and 2B receptors (referred to here as 5-HT_1α _and 5-HT_2β_) [19,20,23]. It has been proposed that Greek letters be used to denote arthropod receptor subtypes in order to distinguish these from vertebrate receptors (classified with Roman letters), emphasizing the fact that the vertebrate and invertebrate subtypes are not orthologous [19]. This convention will be used in the current article.

5-HT_1α _and 5-HT_2β _receptors have been cloned from several crustacean species, including the crayfish *P. clarkii *[19,20,23], and antibodies raised against conserved regions of the orthologous molecules [19,23]. Using these antibodies, the 5-HT_1α _receptor has been localized immunocytochemically in the crayfish brain and ventral nerve cord [23,24]; immunocytochemical localization of the 5-HT_2β _receptor has been reported only in the stomatogastric system [19]. The results presented in the current paper extend these studies by examining the immunocytochemical localization of 5-HT_1α _and 5-HT_2β _receptors as well as the serotonin transporter in the crayfish (*P. clarkii*) brain, and by relating the distributions of these molecules to the expression of receptor mRNAs determined by *in situ *hybridization. Further, we examine in detail the topography of these staining patterns relative to the effects of serotonin on the cell cycle of three generations of neuronal precursors that underlie adult neurogenesis in the crayfish brain.

We have exploited the distinct spatial separation of the neuronal precursor cell generations that produce adult-born neurons in crayfish to define at which level in this lineage serotonin exerts its action. Our results demonstrate that the influences of serotonin on neurogenesis are confined to the late second-generation precursors and their descendants. Further, the presence of 5-HT_1α _and 5-HT_2β _receptor immunoreactivity in these cells, and not in the first- or early second-generation neuronal precursors, indicates that these serotonergic effects are exerted directly on specific generations of cells. Taken together, these results suggest that the influence of serotonin on adult neurogenesis in the crustacean brain is lineage dependent, and that 5-HT_1α _and 5-HT_2β _receptors underlie these effects.

## Results

### Serotonin influences the cell cycle of late second-generation and third-generation neuronal precursors in the *P. clarkii *brain

**Figure 2:**
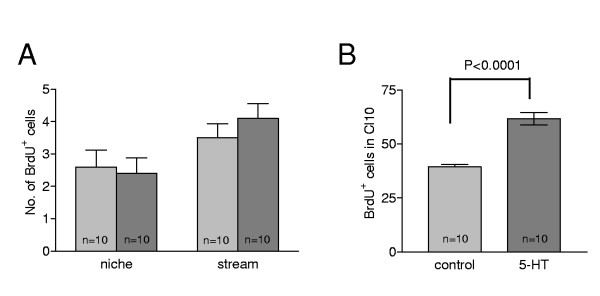
**The influence of serotonin on the lineage of neuronal precursors**. **(A) **5-HT does not alter the number of BrdU-labeled cells in the niches and migratory streams in the brains of treated crayfish (dark gray vertical bar) relative to untreated controls (light gray bar). **(B) **A *t*-test (*P *< 0.0005) reveals significant increases in the number of BrdU-labeled cells in cluster 10 in 5-HT (10^-9 ^M) treated crayfish (*P. clarkii*) compared with untreated controls. Error bars represent standard error of the mean; n = numbers of lateral proliferation zones per group.

The effect of serotonin (10^-9 ^M) exposure *in vivo *on the rate of 5-bromo-2-deoxyuridine (BrdU) incorporation into the neuronal precursor cell lineage was assessed (Figure 2). Serotonin had no impact on the number of BrdU-labeled cells in the niche (first-generation precursors) or migratory streams (second-generation precursors) relative to unexposed control animals (Figure 2A). However, serotonin treatment did increase the numbers of BrdU-labeled cells in the LPZ of cluster 10 (Figure 2B). These data suggest that serotonin does not influence all cells in the neuronal ancestral lineage, but rather that it alters the cell cycle only in the proliferation zones, which are composed of late second-generation precursors and their progeny (Figure 1D).

### Expression pattern of 5-HT_1α _and 5-HT_2β _mRNA in the brains of *P. clarkii*

**Figure 3:**
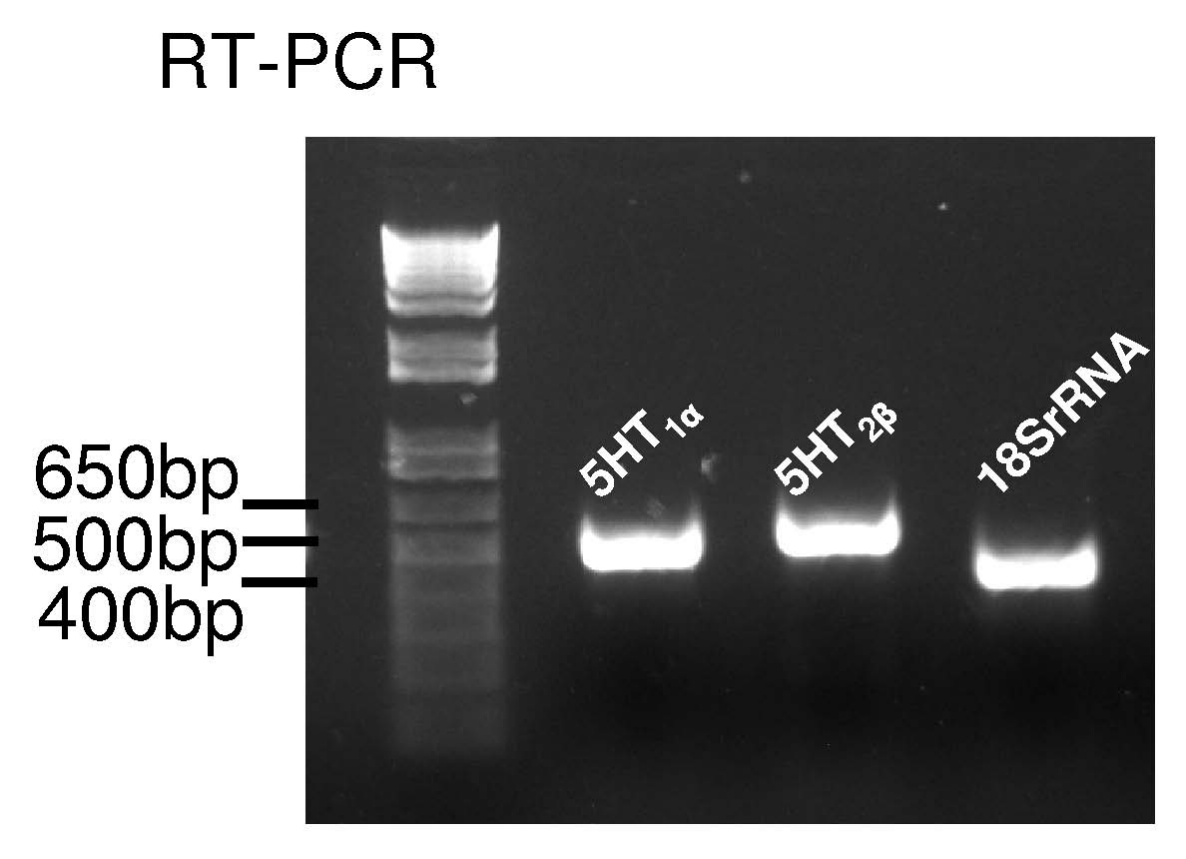
**5-HT receptor mRNAs are expressed in the brain of the crayfish *P. clarkii***. RT-PCR was performed on total RNA sampled from *P. clarkii *brains. The expected PCR products based on GenBank *P. clarkii *sequences are 515 bp for 5-HT_1α _mRNA (lane 2), 546 bp for 5-HT_2β _(lane 3) and 427 bp for 18S rRNA (lane 4). Lane 1, 1-kb DNA ladder.

When total RNA sampled from the brains of crayfish *P. clarkii *was analyzed with RT-PCR, a 515-bp product for 5-HT_1α _(Figure 3, lane 2) and a 546-bp product for 5-HT_2β _(Figure 3, lane 3) were revealed. As an internal control, PCR for 18S rRNA from the same cDNA template was also performed, and a 427 bp product was detected (Figure 3, lane 4). Nucleotide sequence analysis verified that the PCR products correspond to the targeted cDNA fragment of *P. clarkii *5-HT_1α _[GenBank:EU131667] and 5-HT_2β _[GenBank:EU131666].

**Figure 4:**
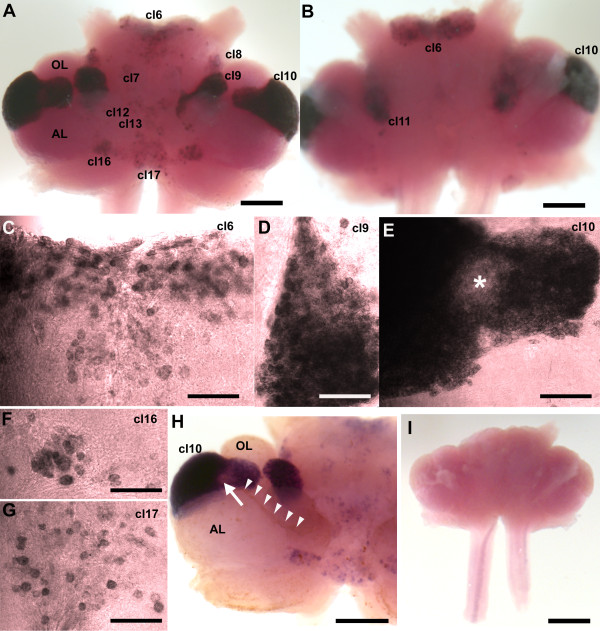
***In situ *hybridization for 5-HT_1α _mRNA in whole mount brains (n = 6) of *P. clarkii***. **(A-H) **Labeling for the 5-HT_1α _transcript with the antisense riboprobe. (A) Ventral view of the brain after hybridization. (B) Dorsal view of the brain after hybridization. Cell clusters (cl) positive for hybridization signals were darkly stained. Selected cell clusters are more highly magnified in (C-G). Intense 5-HT_1α _message labeled with anti-sense riboprobe (purple to black) is found in soma clusters 6 (C), 9 (D), 10 (E), 16 (F), 17 (G), and 7, 8, 11, 12, 13 (not shown). **(I) **The sense probe indicated no detectable signal. (H) In some preparations, a lower 5-HT_1α _expression area was found (arrow) in the area where the glutamine synthetase-labeled migratory stream (brown, indicated with arrowheads just above the stream) inserts into cluster 10. Scale bars: 300 μm (A, B, H); 100 μm (C-G); 500 μm (I). AL, accessory lobe; OL, olfactory lobe. The asterisk in (E) marks the area in cluster 10 with reduced 5-HT_1α _mRNA expression.

*In situ *hybridization with the antisense riboprobe revealed the expression of 5-HT_1α _mRNA in several neuronal cell clusters, but labeling was not detected in fiber tracts or synaptic neuropil regions (Figure 4). Nonspecific hybridization signal was not found, as shown in a comparable brain tested with the sense riboprobe (Figure 4I). In the protocerebrum, cells of cluster 6, which lies at the anterior edge of the median protocerebrum and extends through the brain from dorsal (Figure 4B) to ventral (Figure 4A), contained strong labeling for the 5-HT_1α _transcript (Figure 4C), as did cells in clusters 7 and 8 (Figure 4A). In the deutocerebrum, prominent hybridization signals were found in cells of clusters 9 (Figure 4A, D) and 10 (Figure 4E), the latter of which can be seen in both ventral (Figure 4A) and dorsal (Figure 4B) views of the brain. In some preparations, a region of reduced labeling was found in the middle of cluster 10 (Figure 4E, asterisk). *In situ *hybridization in combination with immunostaining for glutamine synthetase (GS) (Figure 4H, arrowheads), which labels the niche and migratory streams [10], revealed in some preparations that there is a lower 5-HT_1α _expression region (arrow in Figure 4H) at the point where the GS-labeled migratory stream inserts into cluster 10. In addition, cells in deutocerebral clusters 11 (Figure 4B), 12 and 13 (Figure 4A) label with the 5-HT_1α _receptor transcript with varying intensities. In the tritocerebrum, neuronal somata in lateral cluster 16 (Figure 4A, F) and medial cluster 17 (Figure 4A, G) are distinctly labeled.

**Figure 5:**
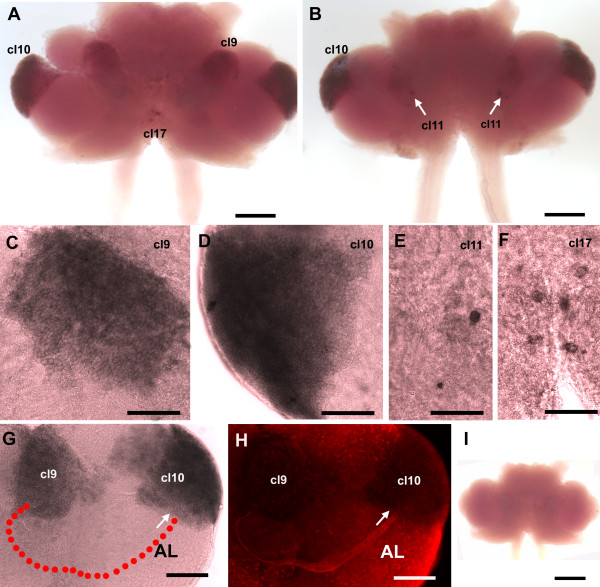
**Whole mount *in situ *hybridization for *P. clarkii *5-HT_2β _(n = 5)**. **(A) **Low magnification ventral view of the brain. **(B) **Low magnification dorsal view of the brain. **(C-F) **More highly magnified views of selected cell clusters (cl). Intense 5-HT_2β _message (purple to black) is found in soma clusters 9 (C), 10 (D), 11 (B, arrows; E) and 17 (F) with antisense riboprobe. **(G, H) **Combined with immunofluoresence for GS (H, red), it is clear that cells in the niche and streams are not labeled (G). The red dotted line is placed just below the outline of the niche and streams that are visualized in (H). Arrows in (G, H) mark the same point where the stream inserts into cluster 10. **(I) **The sense probe indicated no detectable signal. Scale bars: 300 μm (A, B); 100 μm (C-F); 200 μm (G-H); 500 μm (I). AL, accessory lobe.

*In situ *hybridization with the antisense riboprobe for 5-HT_2β _mRNA (Figure 5) revealed an expression pattern similar to 5-HT_1α _mRNA, in that the signal is seen only in cell bodies and not in neuronal fibers or neuropil regions. Overall, labeling for 5-HT_1α _receptor is more prevalent than for 5-HT_2β _receptor, as many cell clusters display 5-HT_1α _but not 5-HT_2β_; this result could be due to differing sensitivities of the probes or to actual differences in abundance of message. The 5-HT_2β _transcript was found mainly in neuronal clusters 9 (Figure 5A, C) and 10 (Figure 5A, B, D). Two pairs of cells in cluster 11 in the medial deutocerebrum were also labeled (Figure 5B, E). In the ventromedial tritocerebrum, four to six cells in cluster 17 consistently expressed the 5-HT_2β _transcript (Figure 5F). Unlike 5-HT_1α _labeling (Figure 4E, H), *in situ *hybridization for 5-HT_2β _did not reveal a lower expression area in the LPZ of cluster 10. Correspondingly, the insertion point of the glutamine synthetase-positive migratory stream in cluster 10 is intensely labeled for the 5-HT_2β _transcript, similar to the other regions of cluster 10 (Figure 5D, G, H). The sense riboprobe showed no nonspecific labeling (Figure 5I).

### 5-HT_1α _protein distribution

**Figure 6:**
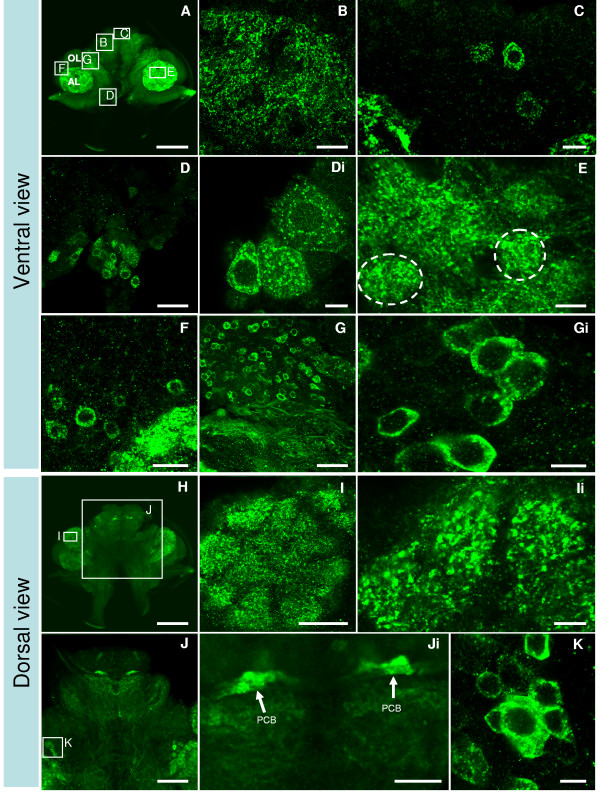
**Immunohistochemistry with antibody specific against *P. clarkii *5-HT_1α _receptor reveals protein expression in cell soma clusters and synaptic neuropil regions (n = 11)**. **(A, H) **Low magnification images show the overall staining pattern in ventral (A) and dorsal (H) views of the brain. **(C-Di, F-Gi, K) **Higher magnification images of staining in clusters 6 (C), 9 (G, Gi), 10 (F), 11 (K) and 16 (D, Di) are also shown. **(B, E, I-Ji) **Intensive 5-HT_1α _protein is also found in neuropils, such as in the anterior (AMPN) and posterior median protocerebral neuropils (PMPN) (B), accessory lobe (AL; (E) with two AL spherical glomeruli circled), olfactory lobe (OL; I, Ii), and protocerebral bridge (PCB; arrows in (J, Ji)). Scale bars: 400 μm (A, H); 100 μm (J); 50 μm (A, C, G, I, Ji); 20 μm (C, F); 10 μm for all other panels.

To define the distribution of the 5-HT_1α _receptor, single and double antibody staining methods were used (Figure 6). In the protocerebrum, strong 5-HT_1crust_-immunopositive terminals are found in lateral extensions of the protocerebral bridge (Figure 6J-Ji). Posterior to the protocerebral bridge, in the anterior and posterior median protocerebral neuropils (AMPNs and PMPNs, respectively), punctate labeling is also found (Figure 6B). Particularly intense staining is found in neuronal terminals that compose conical and spherical glomeruli in the olfactory (OL) (Figure 6I, Ii) and accessory (AL) (Figure 6A, E) lobes, respectively. Strong cytoplasmic staining for 5-HT_1α _is also found in some neuronal somata in several different cell clusters where homogeneous punctate immunoreactivity fills the cytoplasm, leaving the nucleus negatively stained in the middle of the soma: clusters 6 (Figure 6C), 9 (Figure 6G-Gi), 10 (Figure 6F), 11 (Figure 6K), 16 (Figure 6D-Di) and 17 (not shown). Because cytoplasmic labeling in cells is so intense, it is difficult to distinguish membrane-associated labeling; the presence of such distinct cytoplasmic labeling, however, may suggest the presence of newly synthesized or recycled receptor (but see also Discussion).

### Expression of 5-HT_1α _transcript and protein in the neuronal precursor lineage

**Figure 7:**
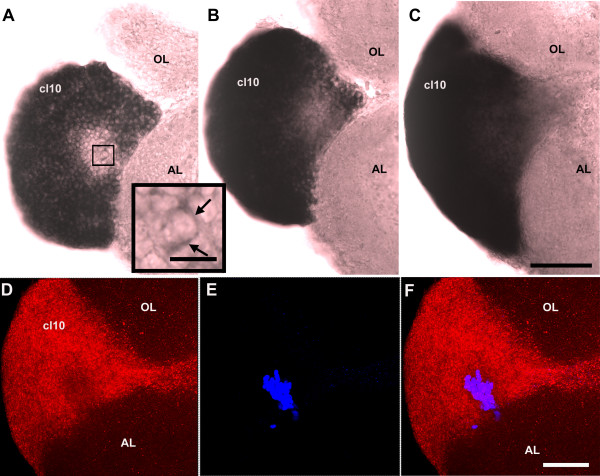
**The region in cluster 10 with reduced 5-HT_1α _mRNA expression is sometimes found deep within the proliferation zone of cluster 10**. **(A-C) **Coronal serial sections (20 μm each) from ventral (A) to dorsal (C) through cluster 10 reveal a relatively low expression area in cluster 10. The inset in (A) shows labeling (arrows) for 5-HT_1α _mRNA within the lower expression region indicated. **(D-F) **Combined with BrdU-labeling, BrdU-positive cells (blue) are found localized in the area of reduced mRNA expression (red). Scale bars: 20 μm (A, inset); 100 μm for all other panels; the scale bar in (C) also applies to (A, B); the scale bar in (F) also applies to (D, E). AL, accessory lobe; cl, cluster; OL, olfactory lobe.

*In situ *hybridization for the 5-HT_1α _transcript revealed a region of reduced expression in cell cluster 10. In some (but not all) preparations, the area of lower 5-HT_1α _expression was superficial and easily seen right after hybridization (Figure 4E, H). In order to precisely localize this region, coronal serial sections (20 μm in thickness) were cut from the post-hybridized brains. Indeed, in the middle of the densely packed cells in cluster 10, some cells were found to express a reduced, but clearly detectable (Figure 7A, inset), level of 5-HT_1α _mRNA in comparison with the surrounding cells (Figure 7A-C, sections from ventral to dorsal). To explore this observation further, *in situ *hybridization for 5-HT_1α _receptor mRNA was combined with BrdU immunofluorescent labeling to reveal the locations of proliferating cells. In some cases, the BrdU-labeled cells (Figure 7E, blue) were positioned in the middle of the reduced 5-HT_1α _expression region (Figure 7D, F, red) in cluster 10.

**Figure 8:**
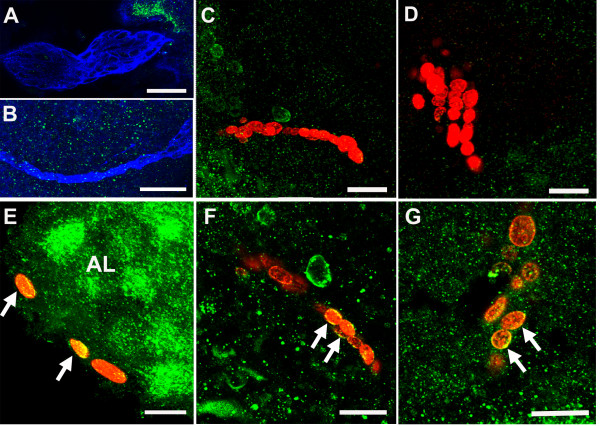
**Double immunofluoresence for 5-HT_1α _(green)/BrdU (red) (n = 6 brains), or 5-HT_1α _(green)/glutamine synthetase (blue) (n = 4 brains), reveals the expression of 5-HT_1α _protein in S phase cells in the distal streams near the proliferation zones, and in the proliferation zones themselves**. **(A, B) **Cells in the neurogenic niche (A) and proximal and medial parts of the migratory streams (B) do not label for the receptor. **(E-G) **Some cells in the distal end of the stream near the proliferation zones (E), as well as cells in the proliferation zones of cluster 9 (F) and cluster 10 (G) show cytoplasmic labeling for the receptor (arrows). **(C, D) **Some BrdU-labeled cells in clusters 9 (C) and 10 (D) are not immunoreactive for the 5-HT_1α _receptor. Scale bars: 30 μm (B); 20 μm (A, C-G). AL, accessory lobe.

Immunocytochemical techniques were also used to ask whether 5-HT_1α _receptor protein is found in neuronal precursor cells in the niche, migratory streams or proliferation zones of clusters 9 and 10. GS labeling reveals the niche (Figure 8A) and streams (Figure 8B); BrdU labeling identifies S phase cells in the streams and proliferation zones (Figure 8C-G). Precursor cells in the neurogenic niche (Figure 8A) and their daughters in the proximal and medial parts of the streams (Figure 8B) do not label for 5-HT_1α _receptor. However, in the distal parts of the streams near the proliferation zones (Figure 8E) and in the proliferation zones (Figure 8F, G), some BrdU-labeled cells show distinct receptor labeling. Occasionally, BrdU-positive cells in the LPZ and MPZ are also seen that are not immunoreactive for the 5-HT_1α _receptor (Figure 8C, D). This variability in receptor labeling among neuronal precursors in the proliferation zones suggests either that serotonin may regulate only a subset of the second/third-generation precursor cell population or that serotonin receptors other than 5-HT_1α _may be present on these cells.

### 5-HT_2β _protein distribution

**Figure 9:**
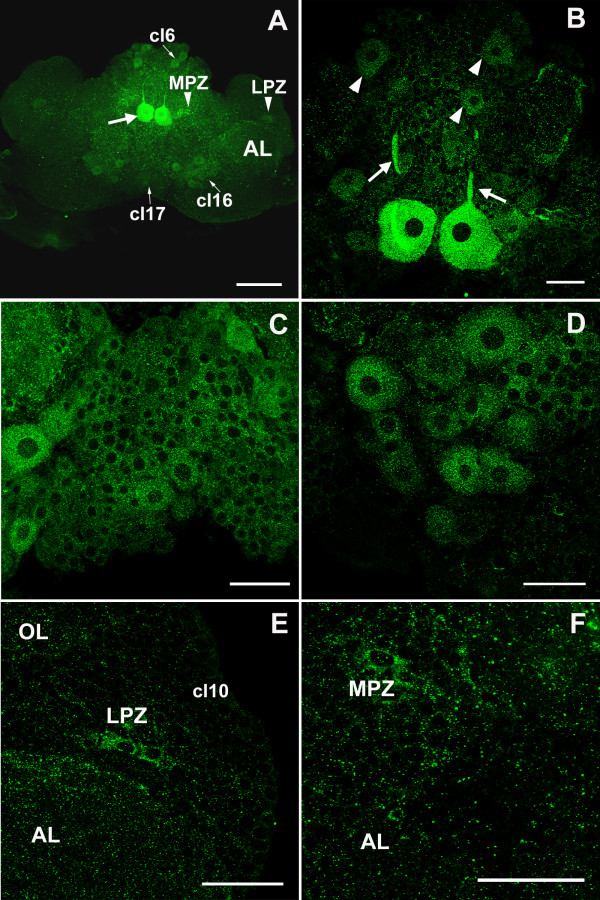
**Immunohistochemistry with antibody specific against *P. clarkii *5-HT_2β _receptor reveals protein expression in some cell soma clusters (n = 14 brains)**. **(A) **Low magnification images show the overall staining pattern seen from the dorsal side. The large arrow points to the paired medial giant neurons, small arrows indicate the locations of clusters 6, 16, and 17, and arrowheads point to the MPZ and LPZ. **(B-F) **Higher magnification images of staining in the medial giant cells and their fibers (B, arrows), cluster 6 (B, arrowheads), cluster 17 (C), cluster 16 (D), the LPZ of cluster 10 (E) and the MPZ of cluster 9 (F). Scale bars: 200 μm (A); 50 μm in the other panels. AL, accessory lobe; cl, cluster; OL, olfactory lobe.

5-HT_2βCrust _immunoreactivity is found in somata in several cell clusters in the crayfish brain (Figure 9), although in most cells this tends to be weaker than labeling observed for the 5-HT_1α _receptor (Figure 6). This could be due to differences in either the affinities of the two antibodies or in the abundance of these two receptors. Similar differential labeling was also observed for 5-HT_1α _and 5-HT_2β _mRNA distribution. Notably, the medial giant cells (Figure 9A) and their long processes (Figure 9B, arrows) projecting toward cluster 6 label intensely with 5-HT_2βCrust_. Cells in clusters 6, 9, 10, 16 and 17 are also labeled. The cells in the LPZ and MPZ are more intensely labeled than other cells in their respective cell clusters (LPZ, cluster 10; MPZ, cluster 9). While immunoreactivity of cells in these regions is consistent, it is relatively weak. Likewise, the olfactory and accessory lobes, which contain projections of somata in clusters 9 and 10, are distinctly, but weakly, labeled (not shown).

**Figure 10:**
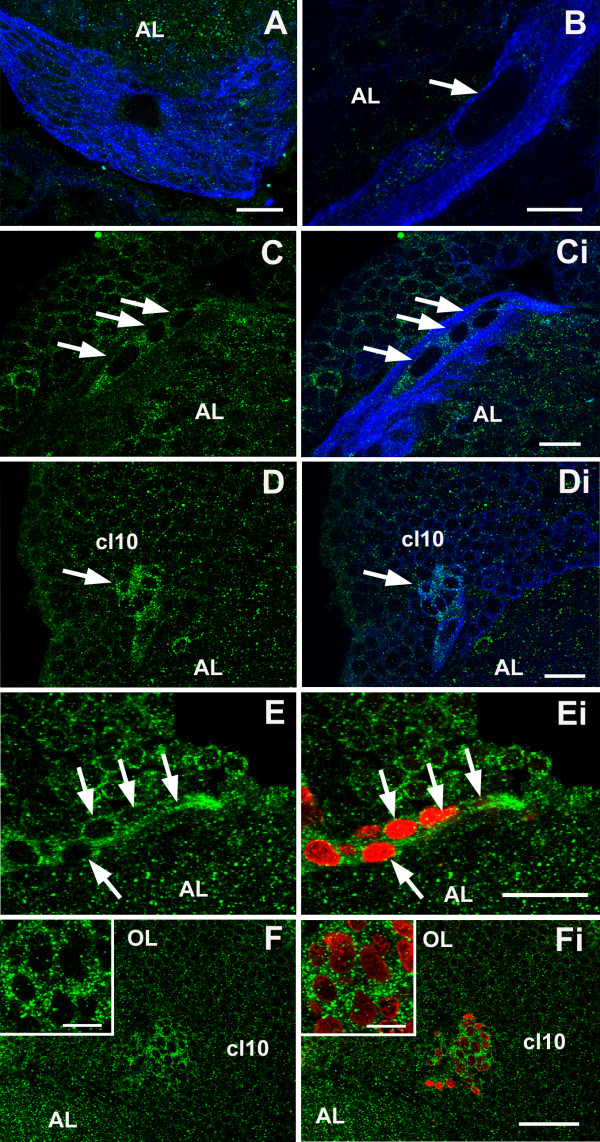
**Double immunofluoresence for 5-HT_2β _(green)/glutamine synthetase (blue) (n = 4 brains) or 5-HT_2β _(green)/BrdU (red) (n = 4 brains) reveals the expression of 5-HT_2β _protein in S phase cells in the distal streams near the proliferation zones, and in the proliferation zones themselves**. **(A, B) **Cells in the neurogenic niche (A) and proximal and medial parts of the migratory streams (B, arrow indicates a GS-positive cell in the proximal part of the stream) do not label for the receptor. **(C-Fi) **Some cells in the distal end of the stream near the proliferation zones (arrows in (C, Ci, E, Ei)), as well as cells in the proliferation zones of cluster 10 (arrows in (D, Di, F, Fi); insets show more highly magnified proliferation zones) show cytoplasmic labeling for the receptor. The left panels (C, D, E, F) show labeling for the 5-HT_2β _receptor; the companion panels on the right (Ci, Di, Ei, Fi) are composites showing receptor immunoreactivity merged with other antibody labels. Scale bars: 50 μm (F, Fi); 10 μm ((B) and insets); 20 μm in the other panels.

Double immunofluorescence for 5-HT_2βCrust _and glutamine synthetase or BrdU revealed the presence of homogeneous cytoplasmic labeling for 5-HT_2β _protein in S phase cells in the distal migratory streams near the proliferation zones (Figure 10C, Ci, E, Ei), as well as in the proliferation zones in clusters 9 and 10 (Figure 10D, Di, F, Fi). However, staining in the LPZ was more intense for 5-HT_2β _than for 5-HT_1α_. In contrast, cells in the neurogenic niche (Figure 10A) and proximal and medial parts of the migratory streams (Figure 10B) do not label for this receptor. The distribution of the 5-HT_2β _protein in the precursor cells is therefore very similar to that observed for the 5-HT_1α _receptor (see above and Figure 8), although the relative labeling intensities for these receptors are sometimes different. For instance, immunocytochemical staining for 5-HT_2β _is stronger in the LPZ than in cluster 10 cells outside the proliferation zone, while the LPZ is more weakly labeled for 5-HT_1α _mRNA than surrounding cluster 10 cells.

### Activation of 5-HT_1α _receptors increases neurogenesis in the LPZ

**Figure 11:**
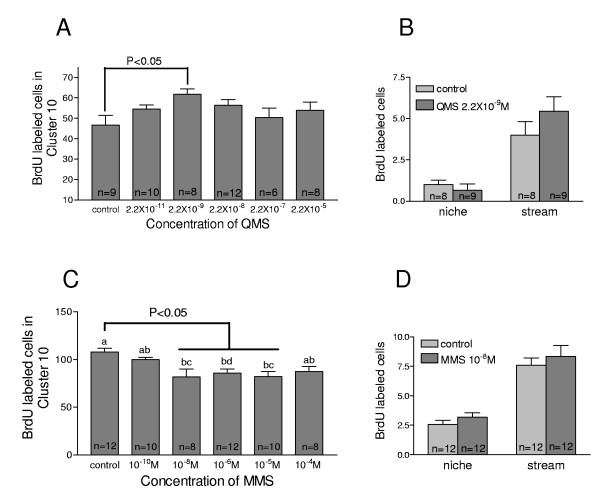
**Pharmacological treatment of serotonin receptors**. **(A) **Dose-response curve for the 5-HT_1α _agonist quipazine maleate salt (QMS) and levels of BrdU incorporation in cells in the LPZ of the crayfish brain. The graph of mean BrdU-labeled cell counts versus QMS concentrations (10^-11 ^to 10^-5 ^M) demonstrates that the rate of cell proliferation in cluster 10 is increased after QMS treatment. One-way ANOVA followed by Tukey multiple comparison (*P *< 0.05) reveals significant differences at QMS levels of 10^-9 ^M relative to control brains. n = number of cluster 10s per group. **(B) **QMS does not alter the number of BrdU-labeled cells in the niches and migratory streams in the brains of treated crayfish relative to untreated controls. **(C) **Dose-response curve for the 5-HT_2β _antagonist methiothepin mesylate salt (MMS) and levels of adult neurogenesis in the LPZ of crayfish brains. The graph of BrdU-labeled cell counts versus MMS concentrations (10^-10 ^to 10^-4 ^M) demonstrates that the rate of cell proliferation in cluster 10 is decreased after blocking the function of 5-HT_2β _with MMS. One-way ANOVA followed by Tukey multiple comparison (*P *< 0.05) reveals significant differences at MMS concentrations of 10^-5 ^to 10^-9 ^M relative to control brains. n = number of cluster 10s per group. Same letter notation on the histograms indicates that there are no statistical differences among the groups; different letters indicate statistical significance. **(D) **MMS does not alter the number of BrdU-labeled cells in the niches and migratory streams in the brains of treated crayfish relative to untreated controls. Error bars represent standard error of the mean.

In order to understand which type of serotonin receptors are involved in the regulation of cell proliferation and neurogenesis after serotonin treatment, the 5-HT_1α _agonist quipazine maleate salt (QMS) was administered. This agonist was chosen because it has been shown to have the highest potency and efficacy in activating 5-HT_1α _receptors from *P. clarkii *compared with nine other pharmacological agents, and because it shows no activity with *P. clarkii *5-HT_2β _receptors [20]. The proliferating cells in cluster 10 were localized using BrdU detection of the nuclei of S phase cells. A gradual increase in the number of BrdU-labeled cells in the LPZ of cluster 10 was observed with increasing QMS concentrations from 2.2 × 10^-11 ^M to 2.2 × 10^-9 ^M (Figure 11A). The activation of 5-HT_1α _receptors with a 10-hour exposure to 2.2 × 10^-9 ^M of QMS induced a significant increase (38%; *P *< 0.05) in the number of BrdU cells relative to controls (Figure 11A). Higher concentrations of QMS did not alter the number of BrdU-labeled cells in cluster 10, a finding that is in agreement with our previous dose-response analysis for serotonin in lobster brains [15].

### Blocking the function of 5-HT_2β _receptors decreases neurogenesis in the LPZ

Experimental animals were treated with different concentrations of the 5-HT_2β _antagonist methiothepin mesylate salt (MMS). MMS was chosen because prior studies [20] demonstrated that this antagonist had the highest efficacy on *P. clarkii *5-HT_2β _receptors among 29 antagonists tested, and no detectable activity on *P. clarkii *5-HT_1α _receptors. In the LPZ in cluster 10, a gradual decrease in BrdU-positive cells was observed with increasing MMS concentrations from 10^-10 ^M to 10^-5 ^M versus controls (Figure 11C). Blocking of 5-HT_2β _receptors for 10 hours with MMS at 10^-8 ^to 10^-5 ^M caused a significant decrease (25%; *P *< 0.05) in BrdU-labeled cells over control levels.

### The influence of 5-HT_1α _and 5-HT_2β _receptors on cell proliferation in the niche and streams

In order to know whether 5-HT_1α _and 5-HT_2β _receptors also influence the numbers of proliferating cells in the niche and migratory streams, we counted the number of BrdU-positive cells in these regions after receptor agonist and antagonist treatment of live animals. In contrast to our findings in the LPZs in cluster 10, neither the 5-HT_1α _agonist QMS (2.2 × 10^-9 ^M) nor the 5-HT_2β _antagonist MMS (10^-8 ^M) caused significant changes in the numbers of BrdU-labeled cells in the niche or stream versus control levels (Figure 11B, D). There is some variability in the baseline number of BrdU-labeled cells in the QMS and MMS treatment groups; these range from one to four BrdU-labeled cells in the niche, and three to ten cells in the streams. We know that the rate of neurogenesis decreases as the animals grow and age [25], and the variability in baseline counts is attributed to the range of animal sizes used. The size of control and experimental animals within each treatment group were, however, tightly controlled. The number of M-phase cells labeled with an antibody against phospho-histone H3 confirms the effects of QMS and MMS on the numbers of dividing cells in the niche, migratory streams and cell cluster 10 (data not shown).

### Serotonin transporter distribution

**Figure 12:**
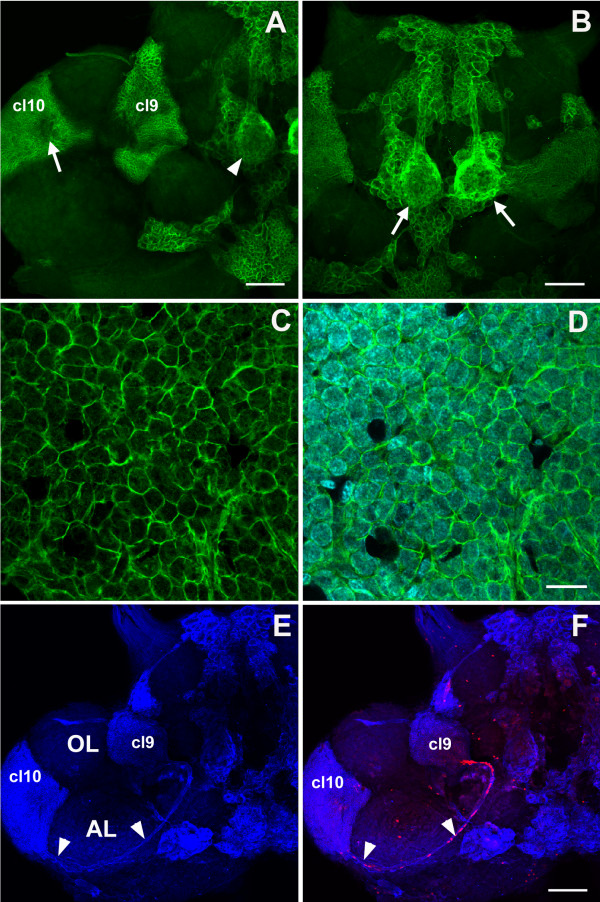
**Immunocytochemical labeling for the serotonin transporter (n = 9 brains)**. **(A-F) **SERT is green in (A-D) and blue in (E-F). Structures with intense labeling for SERT include the cytoplasm of neurons in clusters 9 and 10 (A, C-F), the medial giant neurons (A, arrowhead; B, arrows), and the neurogenic niche and migratory streams (E-F). A lower level of immunoreactivity is seen in the LPZ (A, arrow) relative to surrounding cells in cluster 10, similar to the distribution of 5-HT_1α _mRNA (Figure 7). (C) SERT labeling of cells in cluster 10. (D) DAPI (4',6-diamidino-2-phenylindole, cyan in (D)) labeling of DNA merged with SERT labeling shown in (C). (E) SERT labeling of clusters 9 and 10, and the neurogenic niche and streams (arrowheads). (F) BrdU labeling (red) merged with SERT labeling shown in (E). Scale bars: 200 μm (A, B, E, F); 20 μm (C, D). AL, accessory lobe; cl, cluster; OL, olfactory lobe.

The distribution of serotonin transporter (SERT) also was assessed immunocytochemically (Figure 12). Anti-SERT immunoreactivity is found widely in cells in clusters 6, 9, 10, 11, 16 and 17 (Figure 12A, C, D). Labeling in cluster 10 is particularly intense and appears to be localized to every cell in the cluster (Figure 12C, D), which is composed of 'globuli' cells whose somata are characteristically small and distinct from other brain neurons in that the cell nuclei virtually fill the cell bodies (Figure 12D) [26]. However, as with the localization of 5-HT_1α _mRNA, the LPZ in cluster 10 is more lightly labeled than the surrounding cells (Figure 12A). The medial giant cells, which labeled strongly for 5-HT_2β _receptor, also bind the anti-SERT antibody (Figure 12B). Of particular interest for the present study, the neurogenic system, including the niche cells and their fibers that compose the streams, is intensely labeled (Figure 12E, F).

## Discussion

The data presented here show that serotonin alters the rate of adult neurogenesis in the crayfish brain, that this action is confined to the late second- and third-generation cells that reside in the LPZ of cluster 10, and that these effects are mediated, at least in part, by 5-HT_1α _and 5-HT_2β _receptors. Evidence from several different approaches contribute to these conclusions. First, serotonin increases the numbers of BrdU-labeled cells in the proliferation zones of cluster 10 (as documented previously in the lobster [15] and the Australian crayfish [16]), but does not alter the rate of BrdU incorporation among the niche precursors or their migratory daughters in the streams. Second, RT-PCR shows that 5-HT_1α _and 5-HT_2β _receptors are expressed in the brains of these animals. Third, *in situ *hybridization with antisense riboprobes reveals that both 5-HT_1α _and 5-HT_2β _mRNAs are found in clusters 9 and 10, the only two sites in the crayfish midbrain where adult-born neurons are incorporated. Fourth, immunolabeling with antibodies raised against *P. clarkii *5-HT_1α _and 5-HT_2β _peptides demonstrates that these receptors are absent from the first-generation niche precursor cells and their daughters in the proximal and medial parts of the migratory stream. However, some of the second-generation cells in the distal stream close to the proliferation zones in clusters 9 and 10 do express these receptor proteins; it is not known, however, whether these receptors co-localize to the same cells. These receptor localization data suggest that serotonin acts directly on specific generations in the neuronal precursor lineage, rather than through an indirect pathway. An *in vitro *isolated niche-stream preparation is under development, which will allow a direct test of this idea. Finally, functional assays using a specific 5-HT_1α _agonist (QMS) increases the rate of BrdU incorporation among cells in cluster 10, while the specific 5-HT_2β _antagonist MMS attenuates BrdU labeling in these cells. However, QMS and MMS do not alter the number of BrdU-labeled cells found in the neurogenic niche or in the migratory streams. Additional agonists and antagonists were not tested because QMS and MMS were shown to be superior to other agents in terms of specificity and efficacy on *P. clarkii *5-HT_1α _and 5-HT_2β _receptors [20]. Therefore, in summary, both cytological and functional assays of *P. clarkii *indicate that serotonin acts selectively on the late second-generation neuronal precursors and their descendants in clusters 9 and 10.

### Receptor labeling compared with prior studies

There are similarities and some differences between the immunocytochemical labeling for the 5-HT_1crust _antibody presented here, and the prior findings of Spitzer *et al*. [24] using the same antibody and crayfish species. While labeling is found in the same cell clusters and neuropils as in the earlier study, in our experiments neuropil regions (for example, olfactory and accessory lobes) were most intensely labeled, while in the prior studies cell clusters were most intensely labeled. We also found that immunoreactivity in cell clusters and neuropils was consistent, in contrast to the findings of Spitzer *et al*. [24], where the intensity of labeling with the 5-HT_1crust _antibody, as well as the numbers of cells in the various cell clusters, was highly variable between animals. It was suggested that the variability observed in the prior studies might be 'indicative of the physiological state of the animal' [24]. In this regard the animals used in our assays, for unknown reasons, may have been more uniform. It is more likely, however, that these differences are due to modifications in fixation and processing methods. Spitzer *et al*. also observed an uneven distribution of labeling in the cytoplasm and around the periphery of irregularly shaped somata in the brain; cells showing receptor labeling in our studies were spherical or ovoid, and labeling was homogeneously distributed throughout the cytoplasm. Possible membrane-associated labeling was observed in both studies, a point that requires further investigation at the ultrastructural level.

### Comparison of mRNA and protein expression patterns

The immunocytochemical localization of 5-HT_1α _and 5-HT_2β _receptor protein in the present study identifies sites of serotonin's action in the brain of the crayfish *P. clarkii*. In addition, the *in situ *hybridization results establish which cells synthesize 5-HT receptor proteins and contribute these to the neuropils and to the system generating new neurons in the adult brain. However, the most intense receptor labeling was found in neuropil regions, while 5-HT_1α _and 5-HT_2β _mRNA was predominantly localized to cell somata. For example, we observed intense labeling for the 5-HT_1α _protein, as well as distinct but weak labeling for the 5-HT_2β _receptor, in the olfactory and accessory lobes. These neuropil regions contain axonal fibers and synapses of local and projection interneurons in cell clusters 9 and 10, respectively. However, the mRNA for both 5-HT receptors is primarily found in the somata of cells in clusters 9 and 10 and not in the neuropils.

There are two possible explanations for the distinct intracellular distributions of receptor mRNA and protein. Firstly, this may suggest that there is little or no synthesis of the serotonin receptor protein at the sites where we observe the most intense immunocytochemical labeling. Instead, our data may indicate that these receptors are synthesized in the somata and transported to the synaptic terminals. To date, although local translation of membrane proteins, heat-shock proteins, anti-oxidant proteins at distal axons and their terminals has been reported [27,28], cytoskeletal proteins remain dominant among the axonally synthesized proteins [27]. The slow axoplasmic transport [29] and limited half-life of cytoskeletal proteins [30,31] render local synthesis a more effective means to deliver these proteins to distal axons or growth cones [32,33]. We do not know the half-life of serotonin receptor proteins in crayfish neurons, but the relatively stable lifespan (half-life >100 hours) of 5-HT_1A _and 5-HT_2A _receptors in rat [34] provides a reference. Secondly, therefore, the sparsity of receptor mRNA at sites where protein is most intensely labeled could be an outcome of a relatively short half-life of mRNA compared to the receptor protein. Indeed, in cultured P11 cells derived from rat pituitary tumors, 5-HT_2A _mRNA has an average half-life of only 70 minutes [35]. One does not, therefore, necessarily expect to see co-localization of receptor mRNA and protein.

### Serotonin transporter distribution

Our results and those of Spitzer *et al*. [24] demonstrate intense cytoplasmic labeling for the 5-HT_1α _and 5-HT_2β _receptors. Spitzer *et al*. have suggested that this labeling is likely to represent newly synthesized or recycled receptor. While this is quite possible, an alternative possibility is that at least some of these receptors may be functional as cytoplasmic proteins. One hypothesis is that serotonin may be taken into cells via the serotonin transporter, where it could bind to cytoplasmic receptors to initiate specific functions. It has been proposed that serotonin may act intracellularly as a growth or transcriptional regulator [36-39], although the specific actions are speculative.

To explore the possibility of a cytoplasmic role for these serotonin receptors, we localized sites of SERT labeling in the crayfish brain. Interestingly, the pattern of labeling for the serotonin transporter is similar in many regions to the distribution of receptor labeling. For instance, the medial giant cells that contain intense cytoplasmic labeling for the 5-HT_2β _receptor also label strongly for SERT. Likewise, cells in clusters 9, 10 and 11 contain intense labeling for both the 5-HT_1α _receptor and SERT, while the LPZs are weakly labeled for both 5- HT_1α _and SERT. These similarities between labeling for SERT and cytoplasmic 5-HT_1α _and 5-HT_2β _receptors may suggest a nontraditional role for serotonin in these neurons; serotonin may be taken into cells via the transporter, where it then activates cytoplasmic receptors. The large size of the medial giant cells makes these neurons particularly tractable for testing this hypothesis.

### Functional implications of receptor distribution in the crayfish brain

The distribution of 5-HT_1α _and 5-HT_2β _receptors in somata residing in several cell clusters, as well as in many synaptic neuropils (for example, protocerebral bridge, olfactory and accessory lobes, anterior and posterior median protocerebral neuropils) suggests that serotonin mediates a variety of functions in the crustacean brain. Previous studies have implicated 5-HT_1α _receptors in the different degrees of social dominance in crayfish [40] and in diurnal rhythms in the eyestalk [41], functions that are likely to involve higher order brain pathways. Our studies and those of Spitzer *et al*. [24] suggest a possible role for 5-HT_1α _receptor in processing of olfactory information, as well as in higher order integrative functions mediated by the accessory lobes [8,9]. Less is known about possible physiological actions of 5-HT_2β _receptor, although these have been localized to the processes and somata of lateral giant neurons, which are involved in the tail-flip escape response. Our present studies demonstrate the presence of 5-HT_2β _receptor immunoreactivity in the cytoplasm of the medial giant cells, which also are part of the tail-flip circuitry [42,43].

The primary goal of our studies was to examine the distribution of 5-HT_1α _and 5-HT_2β _receptors in the lineage of precursor cells that is responsible for the production of neurons in the adult crayfish brain, and to relate these findings to the effects of serotonin on the cell cycle of each generation of precursors. Exposure of intact animals to serotonin increases BrdU incorporation in the LPZ, which contains the late second- and third-generation neuronal precursors, but not into the first-generation precursors or their migrating daughters. This lineage will produce neurons that will differentiate into projection neurons that innervate the olfactory and accessory lobes. Pharmacological experiments with the 5-HT_1α _receptor agonist QMS and the 5-HT_2β _receptor antagonist MMS are consistent with the effects of serotonin on neuronal precursors, and with the distribution of these receptors in the neurogenic lineage.

### Serotonin and neurogenesis in crustaceans and mammals

These data are of additional interest because many features of adult neurogenesis are evolutionarily conserved. In mammalian and decapod crustacean brains, new neurons are added throughout life to the primary olfactory processing areas. The first generation neuronal precursors, which have glial properties, are located in specialized vascularized niches; their daughters migrate to sites where they will proliferate again and their progeny will differentiate into neurons [10,44-46]. Further, the timing and rate of proliferation in the adult crustacean brain, as in the mammalian brain, are influenced by circadian signals, age, diet, environmental enrichment, nitric oxide and serotonin [11,47].

Serotonin is a particularly potent regulator of neurogenesis during both embryonic and adult life in mammals [48-51] and crustaceans [14-16]. Depletion of brain serotonin results in a decrease in production of new neurons in the dentate gyrus and the subventricular zone of adult rats [4], and in clusters 9 and 10 containing local and projection interneurons in the olfactory pathway of lobsters [3,14]. The general influence of serotonin in the regulation of neurogenesis is therefore conserved across species.

The effects of serotonin on neurogenesis are mediated via its receptors. In mammals, several receptor subtypes are involved in regulating neuronal proliferation in the subgranular zone of the hippocampus and the subventricular zone that contributes new neurons to the olfactory bulb. By various accounts, the activation of 5-HT_1A _and 5-HT_2C _receptors increases neurogenesis in the subventricular zone/olfactory bulb, while 5-HT_1A, _5-HT_1B_, 5-HT_2_, 5-HT_2A_, and 5-HT_2C _receptors have been implicated in diverse effects on neuronal proliferation in the subgranular zone/hippocampus [52-58]. While it is apparent that there are differential actions of these receptors during distinct phases of neurogenesis in these brain regions, methodological disparities and contradictory results hinder clarity regarding these events. The interpretation of 5-HT receptor actions in mammalian systems also have been complicated by the complexity of the precursor cell lineage that produces adult-born neurons, the fact that multiple precursor generations coexist in neurogenic niches, and the relative scarcity of type 3 cells, which are a key population [55]. These challenges underscore the potential value of addressing the fundamental question of lineage-dependent influences of serotonin using the crayfish model, where precursor cell generations are spatially separated and quantitative changes are easily assessed. Furthermore, the use of diverse species to address important questions about adult neurogenesis is likely to result in a broader understanding of specific issues, and of how evolutionary processes may have shaped the vertebrate/mammalian condition.

In the crayfish brain, 5-HT_1α _and 5-HT_2β _receptors first appear in the late second-generation neuronal precursors at the end of their tangential migration across the ventral surface of the brain, as they reach the proliferation zones where olfactory interneurons will proliferate and differentiate. Neither the first generation precursor cells in the niche nor their daughters in the proximal and medial parts of the migratory stream express 5-HT_1α _receptor. Further, the level of 5-HT_1α _mRNA expression in the proliferation zones appears to be lower than in the mature cluster 10 neurons. This pattern suggests that after the first expression of 5-HT_1α _receptor in the late second-generation precursors, receptor expression increases as their progeny differentiate into neurons. The functional assays using the 5-HT_1α _agonist QMS corroborate the receptor localization, as QMS upregulates BrdU incorporation only in the proliferation zones of cluster 10, not among the niche precursors or in their daughters during migration to the proliferation zones. The story with the 5-HT_2β _receptor appears very similar, in that 5-HT_2βCrust _immunoreactivity is confined to the late second- and third-generation cells in the distal migratory streams and proliferation zones of cell clusters 9 and 10, as is the action of the 5-HT_2β _antagonist MMS on cell proliferation in this system. This sequence of events is reminiscent of a study of the maturation of precursor cells migrating in the rostral migratory stream, which shows temporal and spatial regulation in the appearance of GABA and glutamate receptors, with GABA_A _receptors expressed first in the tangentially migrating class 1 cells [59], followed by AMPA receptors [60]; during the next phase of maturation, the radially migrating class 2 cells express NMDA receptors.

Another interesting aspect of the 5-HT_1α _receptor labeling pattern is the finding that only some of the cells in the distal migratory stream express the receptor. This variability may reflect heterogeneity in the spatiotemporal expression of the receptor in different cells, with the possible end point that all second-generation precursors will contain 5-HT_1α _receptor. Alternatively, other classes of 5-HT receptors that were not assessed in this study may be involved in serotonin's action on cells in the proliferation zones. Finally, it may be that only a portion of the late second-generation precursors are sensitive to serotonin. It is known that there are at least two types of local interneurons in cluster 9 expressing either the neurotransmitter orkokinin or allatostatin-like peptide [10]. Similarly, there are two functional categories of projection neurons, those that innervate the olfactory lobe and others that innervate the accessory lobe [13,61]. The presence or absence of serotonin receptors in the late second-generation precursors may therefore reflect distinctive chemical/hormonal sensitivities involved in diverging cell fates.

## Conclusions

While the expression of 5-HT receptors in different cell clusters beyond those involved in adult neurogenesis suggests that serotonin and its receptors serve multiple functions in the crayfish brain, our experiments strongly suggest that serotonin receptor subtypes 1α and 2β participate in serotonin-induced neurogenesis in the brain of decapod crustaceans. Further, the expression and action of these receptors indicate that the influence of serotonin on adult neurogenesis in the crayfish brain is lineage dependent.

## Methods

### Animals and tissue processing

Freshwater crayfish (carapace length 4 to 20 mm) *P. clarkii *(Malacostraca, Decapoda, Astacidae) of both sexes were obtained from Carolina Biological Supply Company (Burlington, NC, USA) and maintained at 21°C in aquaria with recirculating artificial pond water and a light:dark cycle of 12:12 hours. Because the basal rate of neurogenesis decreases with increasing age/size [25], within each experiment the animals were carefully size-matched to within 1 to 2 mm carapace length.

### Serotonin administration and BrdU labeling

Experimental animals were treated with serotonin creatinine sulfate (10^-9 ^M; Sigma, St Louis, MO, USA, no. H7752) and BrdU (2 mg/ml; Sigma, no. B5002) in pond water for 18 hours before sacrificing. Control animals were incubated in pond water with BrdU only (2 mg/ml) during the same time period. BrdU is incorporated into the DNA of replicating cells during the S phase of the cell cycle and can be visualized immunocytochemically [62]. The dose of serotonin used was based on previous results, as was the duration of application [15]. The exposure time was determined by the window required to observe changes in the proliferation of neuronal precursors.

### Pharmacological administration of QMS and MMS

To determine whether 5-HT_1α _and 5-HT_2β _receptors are involved in the serotonergic regulation of cell proliferation in the brains of *P. clarkii*, live animals were treated separately with QMS (a specific *P. clarkii *5-HT_1α _agonist; Sigma, Q1004), or MMS (a specific *P. clarkii *5-HT_2β _antagonist; Sigma, M149) for 2 hours, then with a mixture of QMS or MMS together with 2 mg/mL BrdU in pond water for another 8 hours before sacrificing. These pharmacological agents were selected based on previous studies of their superior specificity and efficacy on *P. clarkii *5-HT_1α _and 5-HT_2β _receptors [20]. They were applied to intact animals in order to avoid artifacts associated with *ex vivo *systems. Control animals of the same size were treated with only BrdU for 8 hours.

### RT-PCR

Total RNA was extracted from crayfish tissues with TRIzol reagent according to the manufacturer's instructions (Invitrogen™ Life Technologies, USA). Reverse transcription was performed following the RNA extraction, and first-strand cDNA was synthesized with a random hexamer and SuperScript III reverse transcriptase (Invitrogen). PCR amplification of cDNA was performed using REDTaq ready mix PCR reaction mix (Sigma, no. R2523). The forward and reverse primers of 5-HT_1α_, 5-HT_2β, _and 18S ribosomal RNA (18S) were designed based on published sequences for *P. clarkii *[GenBank:EU131667, EU131666, and AF436001]. These were: 5'-AGAACACGACGAGCGATGA-3' and 5'-GCCAAGAATGACGGAAGTAA-3' (5-HT_1α_); 5'-GATCTGTCCGCTGGAAGAAG-3' and 5'-ACCTGAAGCTCGAGTCGTGT-3' (5-HT_2β_); and 5'-CTTCTTAGAGGGATTAGCGG-3' and 5'-TACGGAAACCTTGTTACGACTT-3' (18S).

### cRNA probes

Sense and anti-sense specific cRNA probes (riboprobes) for both 5-HT_1α _and 5-HT_2β _receptors were generated by first preparing modified PCR products similar to those described above where forward and reverse 5-HT receptor-specific primers also contained RNA polymerase initiation site sequences for T3 and T7 RNA polymerase, respectively. These modified PCR products were then used as templates, together with a DIG-labeling kit (Roche Molecular Biochemicals, Mannheim, Germany), to generate sense riboprobes with T3 RNA polymerase or anti-sense riboprobes with T7 RNA polymerase.

### *In situ *hybridization

For whole mount *in situ *hybridization, dissected brains were fixed overnight at 4°C (4% w/v paraformaldehyde in RNAse-free 0.1 M phosphate buffer (PB; 20 mM NaH_2_PO_4_, 80 mM Na_2_HPO_4_; pH 7.4), and washed with PBTx (RNAse-free PB with 0.3% Triton X-100). In some cases endogenous peroxidase activity was quenched with 2% H_2_O_2 _treatment for 20 minutes. The brains were then acetylated in 0.1 M triethanolamine/acetic anhydride for 15 minutes, rinsed several times in PB and then in 2× SSC (SSC = 0.15 M NaCl, 0.015 M Na-citrate) for 5 minutes each. They were subsequently submerged for at least 16 hours at 70°C in hybridization buffer (50% formamide (Invitrogen), 5× SSC, 2% Blocking Reagent (Roche, no. 11096176001), 0.02% SDS, and 0.1% N-laurylsarcosine) with riboprobe concentrations of approximately 20 ng/ml. Sequential post-hybridization washes were performed at 70°C using hybridization buffer, 2× SSC, and finally 0.2× SSC. Whole mounts were then blocked in 0.1 M maleate buffer (pH 7.0) containing 1% Blocking Reagent and incubated in sheep anti-DIG-Fab fragments conjugated to alkaline phosphatase (0.15 U/ml; Roche, no. 11093274910) in maleate/blocking buffer for 1.5 hours. After rinses in maleate buffer and then in 0.1 M TRIS, 0.15 M NaCl, pH 9.5, brains were developed in the dark in substrate solution (pH 9.5) containing 0.1 M TRIS, 0.15 M NaCl, 50 mM MgCl_2_, 2 mM levamisole, 0.4 mM 5-bromo-4 chloro-indolylphosphate (BCIP) and 0.4 mM nitroblue tetrazolium chloride (NBT) (both from Roche) to yield a dark purple color. Development time ranged from 4 to 13 hours until the desired color was achieved. Brains were rinsed in PB and images captured with a stereomicroscope (Nikon SMZ1500) or flat mounted in Clear-Mount (EMS, Hatfield, PA, USA) and then captured with a Nikon Eclipse 80i. Some post-hybridization brains were further immunolabeled by incubation with mouse anti-GS (1:100; BD Biosciences Pharmingen, San Jose, CA, USA, no. 610517) followed by goat anti-mouse IgG-HRP (1:600; Jackson Immunoresearch, West Grove, PA, USA, no. 115-035-166) or donkey anti-mouse IgG-Cy3 (1:100; Jackson Immunoresearch, no.711-165-152). HRP immunolabeling was visualized with the peroxidase substrate diaminobenzidine (DAB) (Sigma). In some cases post-hybridization brains were cryoprotected in 30% sucrose followed by cryosectioning into 20 μm sections.

For *in situ *hybridization in combination with BrdU immunofluoresence, fluorescence *in situ *hybridization was performed on whole mount brains. Following the blocking step in TRIS-NaCl buffer containing 0.5% blocking reagent (TNB; PerkinElmer, Boston, MA, USA) for 30 minutes at 21°C, the post-hybridization brains were incubated for 16 hours at 4°C in anti-DIG-POD (1:50 in TNB; Roche, no. 11207733910). Subsequently, brains were rinsed in TNT (0.1 M TRIS, 0.15 M NaCl, 0.05% Tween-20), and incubated for 1 hour in Cy3-tyramide (1:50; PerkinElmer, SAT704A). Brains were then incubated sequentially with mouse anti-BrdU antibody (1:50 in TNB; BD Biosciences, no. 34758) and then with Cy5-conjugated donkey anti-mouse IgG secondary antibody (1:100 in TNB; Jackson Immunoresearch, no. 715-175-151). The brains were rinsed in TRIS-NaCl and coverslips were applied with Fluoromount (EMS). Control brains were hybridized with sense riboprobes.

### Immunohistochemistry

For immunocytochemistry, brains were dissected in cold crayfish saline (205 mM NaCl, 5.4 mM KCl, 34.4 mM CaCl_2_, 1.2 mM MgCl_2 _and 2.4 mM NaHCO_3_) and then fixed (4% w/v paraformaldehyde in PB) for 16 hours at 4°C. Immunofluorescence methods for 5-HT_1α _and 5-HT_2β _receptors were modified from previously published protocols [19,23]. In brief, fixed brains were dehydrated through an ethanol series (10 minutes each: 30, 50, 70, 80, 90, 95, 100%), then rehydrated (100, 70, 50, 30% ethanol). Preparations were washed in PBTx and then incubated 16 hours at 4°C with 2 μg/ml rabbit anti-5-HT_1α _(5-HT_1crust _[19,24]) or with 0.5 to 1 μg/ml rabbit anti-5-HT_2β _(5- HT_2βCrust _[19]) antibody (gift from D Baro, Department of Biology, Georgia State University). After a 2-hour wash in PBTx, the brains were incubated in donkey anti-rabbit IgG-Cy2 (1:100 in PBTx; Jackson Immunoresearch, no. 711-225-152) for 16 hours at 4°C. The preparations were then washed in PBTx, dehydrated sequentially in a graded ethanol series up to 100%, cleared in methyl salicylate (Sigma, M-6752) and mounted in DPX (Fluka Chemie, Buchs, Switzerland, no. 44581). The specificity of the primary antibody for 5-HT_1crust _was confirmed by incubating brains with antiserum that was preadsorbed with the peptide KDPDFLVRVNEHKKCLVSQD (gift from D Baro, Department of Biology, Georgia State University) which was used to raise the antibody. The primary antibody for 5- HT_2βCrust _was raised against peptide DRFLSLRYPMKFGRHKTRRR and its specificity was confirmed by Clark *et al*. [19] with antiserum that was preadsorbed with this peptide.

For double labeling of 5-HT_1α _or 5-HT_2β _receptors and BrdU, the fixed brains from BrdU-treated animals were first treated with 2N HCl for 45 minutes, and then a mixture of 5-HT_1crust _or 5- HT_2βCrust _and mouse anti-BrdU (1:50; BD Biosciences) was applied. The secondary antibodies were donkey anti-rabbit IgG-Cy2 and donkey anti-mouse IgG-Cy3 (1:100). For double labeling of 5-HT_1α _or 5- HT_2β _receptors and GS, the brains were sequentially incubated first with a mixture of rabbit 5-HT_1crust _or 5- HT_2βCrust _and mouse anti-GS, and then a mixture of donkey anti-rabbit IgG-Cy2 and donkey anti-mouse IgG-Cy5. The GS antibody served as a glial marker, as reported in previous studies in crustaceans [10,63,64].

For labeling of SERT, an antigen retrieval step of 2N HCl for 45 minutes was added before incubation in mouse monoclonal anti-SERT antibody (1:1000; Advanced Targeting Systems, San Diego, CA, USA, no. AB-N09). The retrieval step significantly increases the signal to noise ratio. The immunogen was a peptide from the fourth extracellular domain of rat SERT. The specificity of anti-SERT was confirmed by preadsorption with peptide (CEMRNEDVSEVAKDA; Advanced Targeting Systems, no. PR-03). For double-labeling with BrdU, rat anti-BrdU (Accurate Chemical Co., Westbury, NY, USA, no. OBT0030G) with donkey-anti-rat IgG-Cy3 (Jackson Immunoresearch, no. 712-165-153) was used.

### Microscopy and image analysis

Before mounting brains, color images of the *in situ *hybridizations were captured with a stereomicroscope (Nikon SMZ1500). After mounting, the preparations were photographed with a Nikon Eclipse 80i microscope to obtain highly magnified images. Fluorophore-labeled specimens were visualized with a Leica TCS SP laser scanning confocal microscope equipped with argon 488 nm, krypton 561 nm and helium-neon 633 nm lasers. Serial optical sections were taken at intervals of 1 μm and saved as both three-dimensional stacks and two-dimensional projections. Image preparation, assembly and analysis were performed in Photoshop 7 (Adobe Systems, San Jose, CA, USA). Only the color balance and contrast of the images were adjusted.

### Data analysis and statistics

The numbers of BrdU-labeled cells in the niche, migratory streams and the LPZs in cluster 10 were blind counted by individual observers as previously described [25]. In brief, a single optical section was projected onto the monitor and the labeled cells traced onto a transparent sheet. This was repeated for each optical section and the cell profiles then counted from the sheets. All data are presented as mean ± standard error of the mean. Comparisons between different groups of animals were made with Student *t*-tests, or one-way ANOVA analysis followed by Tukey's multiple comparison tests, using Prism software (GraphPad Software, San Diego, CA, USA). Labeled cells in cluster 9 were not assessed quantitatively in the present studies because the MPZ of cluster 9 is less well-defined than the LPZ in cluster 10. The proliferation zone that contributes cells to cluster 9 (MPZ) is located slightly posteromedially from cluster 9 and merges with migratory cells, while the proliferation zone contributing cells to cluster 10 (LPZ) is highly localized within cluster 10 (see Figure 1), providing a more reliable quantitative assay.

## Abbreviations

5-HT: 5-hydroxytryptamine (serotonin); AL: accessory lobe; bp: base pair; BrdU: 5-bromo-2-deoxyuridine; GS: glutamine synthetase; LPZ: lateral proliferation zone; MMS: methiothepin mesylate salt; MPZ: medial proliferation zone; OL: olfactory lobe; PB: phosphate buffer; QMS: quipazine maleate salt; SERT: serotonin transporter.

## Competing interests

The authors declare that they have no competing interests.

## Authors' contributions

YZ conceived of the study, designed and executed most of the experiments, and participated in data interpretation and manuscript preparation. JLB conducted the experiment described in Figure 2, and provided technical support for other experiments. BSB was involved in the overall design of the study, data interpretation and manuscript preparation. All authors have read and approved the final manuscript.
